# Associations Among Physical Exercise, Social Support, and Meaning in Life in College Students: An Actor–Partner Interdependence Model

**DOI:** 10.3390/bs16071056

**Published:** 2026-06-25

**Authors:** Baole Tao, Zhenwu Li, Jie Han, Tianci Lu, Hanwen Chen, Jun Yan

**Affiliations:** College of Physical Education, Yangzhou University, Yangzhou 225012, China; mz120250810@stu.yzu.edu.cn (Z.L.); mz120250783@stu.yzu.edu.cn (J.H.); dx120240094@stu.yzu.edu.cn (T.L.); dx120230091@stu.yzu.edu.cn (H.C.); yanjun@yzu.edu.cn (J.Y.)

**Keywords:** physical exercise, social support, meaning in life, college students, actor–partner interdependence model (APIM)

## Abstract

Background: Physical exercise is known to promote positive psychological functioning among college students; however, its relationship with meaning in life within naturally occurring friendship dyads remains underexplored. This study examined both actor and partner associations between physical exercise, social support, and meaning in life. Methods: A cross-sectional dyadic survey was conducted among 415 friendship dyads (830 Chinese college students, aged 17–23 years). Participants completed validated measures of physical exercise, perceived social support, and meaning in life. Intraclass correlations, Pearson correlations, actor–partner interdependence models (APIMs), and an indistinguishable APIM mediation model were applied to assess within-dyad nonindependence, actor and partner effects, and indirect pathways through social support. Results: Intraclass correlations revealed within-dyad similarities in physical exercise, social support, and meaning in life, with ICCs of 0.101, 0.188, and 0.253, respectively. The constrained indistinguishable mediation model demonstrated acceptable fit: χ^2^(6) = 12.81, *p* = 0.046; CFI = 0.973; TLI = 0.933; RMSEA = 0.052; SRMR = 0.028. Physical exercise was positively associated with social support at both the actor level (B = 0.150, *p* < 0.001) and partner level (B = 0.095, *p* < 0.001). Social support, in turn, was positively linked to meaning in life at both actor (B = 0.174, *p* < 0.001) and partner levels (B = 0.206, *p* < 0.001). Direct effects of physical exercise on meaning in life remained significant for both actor (B = 0.055, *p* = 0.001) and partner pathways (B = 0.067, *p* < 0.001). Bootstrap analyses confirmed significant total indirect effects for actors (B = 0.046, 95% CI [0.030, 0.063]) and partners (B = 0.047, 95% CI [0.032, 0.065]). Conclusions: Physical exercise is associated with meaning in life not only via intrapersonal pathways but also through interpersonal pathways within friendship dyads. Social support serves as a key mediating factor in this relationship.

## 1. Introduction

In recent years, the mental health of college students has emerged as a critical public health concern globally. Recent epidemiological data from China show that suicide-related behaviors among adolescents are increasingly observed in younger age groups, with prevalence rising from 16.3% in 2014 to 18.5% in 2023 (Dong et al., 2025). This trend may reflect broader psychosocial challenges, including a diminished sense of meaning in life. Empirical studies indicate that meaninglessness is relatively prevalent among college students, and is associated with existential confusion, diminished self-worth, and hopelessness regarding the future (Gai et al., 2016; Gao et al., 2025; Tang, 2023).

Meaning in life generally refers to individuals’ understanding and experience of their existence, life goals, and life value (Steger et al., 2006). Classic measurement studies have distinguished meaning in life into dimensions such as presence of meaning and search for meaning, providing an important methodological foundation for subsequent empirical research. In a study of 3196 college students, Guo et al. (2024) reported that meaning in life reflects individuals’ ability to understand their existence and recognize their life goals and tasks. They further found that physical exercise was not only directly associated with meaning in life, but also indirectly associated with it through self-efficacy and life satisfaction. Similarly, Zhao and Yin (2024) found, based on a sample of 923 Chinese college students, that physical exercise was significantly associated with meaning in life, self-concept, and self-control, and that self-concept and self-control played a sequential mediating role between physical exercise and meaning in life. Taken together, these findings suggest that physical exercise may be associated with a more positive experience of meaning in life, although such associations may depend not only on exercise amount but also on its social and experiential context.

Despite this evidence, existing studies on the relationship between physical exercise and meaning in life have largely explained this association through psychological processes within the individual, such as self-efficacy, self-control, self-concept, and life satisfaction (Zhao et al., 2025). Although these studies have provided important evidence for understanding the positive psychological functions of exercise, they have paid relatively limited attention to the interpersonal contexts in which physical exercise occurs. The present study argues that physical exercise should be understood not only as an individual health behavior, but also as a relationally embedded activity that may connect friends through shared contexts, mutual encouragement, perceived support, and joint meaning-making. In everyday college life, exercise behaviors often occur in social settings, including physical education classes, club activities, dormitory life, exercise with friends, and campus leisure activities (Eime et al., 2013; Z. Zhou et al., 2024). Eime et al.’s systematic review showed that physical exercise participation has psychological, psychosocial, and social health benefits, and that team or club sports may be more beneficial to psychosocial health than individual activities due to their social attributes (Eime et al., 2013). Thus, examining the role of physical exercise in promoting mental health from a relational perspective may better reflect college students’ everyday experiences than explanations based solely on individual psychological variables (Eime et al., 2013; Li & Huang, 2025).

### 1.1. Social Support, Physical Exercise, and Meaning in Life: Theoretical Framework

Social support provides an important theoretical bridge linking physical exercise and meaning in life. According to the stress-buffering and main-effect perspectives of social support, supportive relationships may benefit psychological functioning by providing emotional, informational, and instrumental resources in both stressful and everyday contexts (Cohen & Wills, 1985). Thoits (2011) further emphasized that social support contributes to well-being through emotional sustenance, behavioral guidance, identity validation, and a sense of belonging. These mechanisms are particularly relevant for college students, whose daily behaviors, emotional experiences, and self-understanding are strongly embedded in peer relationships.

Social support may be closely related to engagement in physical exercise for several reasons. First, close friends can provide encouragement, companionship, and behavioral modeling, which may make exercise participation more accessible and psychologically rewarding. Second, exercise settings, including physical education classes, campus sport clubs, dormitory-based activities, and leisure-time exercise, create repeated opportunities for support exchange and relational reinforcement. Third, when exercise is embedded in friendship contexts, it may become more than an individual health behavior; it may also serve as a shared social activity through which peers establish routines, affirm one another’s efforts, and strengthen relational belonging. Thus, social support is theoretically relevant to physical exercise because it can shape both the motivation for participation and the interpersonal meaning attached to exercise experiences. Social support may also contribute to meaning in life. Supportive social relationships provide individuals with acceptance, belonging, and value affirmation, thereby helping them integrate everyday experiences into psychological narratives characterized by direction, coherence, and purpose. In close friendship dyads, friends may help one another interpret challenges, affirm personal value, and maintain a sense of future orientation.

Recent studies have shown that physical exercise is closely associated with perceived social support, self-esteem, resilience, and subjective well-being. For example, in a study of 846 college students, Pan et al. (2025) found that physical exercise had significant positive effects on perceived social support, self-esteem, and resilience, with social support and self-esteem mediating the relationship between physical exercise and subjective well-being. Similarly, a study on physical exercise and social adaptation among college students found that physical exercise was significantly and positively associated with social adaptation, self-esteem, and peer attachment, and that peer attachment contributed to the mediating process between physical exercise and social adaptation (Z. Zhou et al., 2024). Taken together, these findings suggest that physical exercise may promote positive psychological development among college students by enhancing peer attachment, social support, and social adaptation.

Stable theoretical and empirical links have been established between social support and meaning in life (Lambert et al., 2010; Thoits, 2011). Recent studies of Chinese college students and vocational undergraduates have shown that perceived social support, meaning in life, and depressive symptoms are closely associated (Peng, 2025). Network analysis studies have further identified meaning in life and friend and family support as important nodes for understanding networks of depressive symptoms (Zhang et al., 2025). Longitudinal studies have also shown that perceived social support can predict depressive levels among college students through gratitude and meaning in life. Therefore, within the framework of sports promoting mental health development, social support should not be regarded merely as a background variable. Instead, it should be understood as an important relational resource linking physical exercise and positive psychological outcomes. In friendship dyads, perceived social support may be particularly important because it is not only experienced by individuals but also exchanged, reinforced, and interpreted within ongoing peer interactions.

### 1.2. Dyadic Interdependence and the Actor–Partner Interdependence Mediation Model

Nevertheless, current research still has a key limitation: most studies use the individual as the basic unit of analysis, assume that each participant is independent, and rarely examine the interdependence among physical exercise, social support, and meaning in life within college students’ actual close peer relationships (Kenny et al., 2006; Ledermann et al., 2011). For college students, close friends are not only an important source of social support, but also a crucial relational context for daily behavioral choices, emotional experiences, and meaning construction. Accordingly, the key gap addressed in this study is whether the association between physical exercise and meaning in life extends beyond individual psychological benefits to dyadic interdependence within close friendship pairs. That is, the present study does not simply ask whether students who exercise more report greater social support and meaning in life; rather, it asks whether these associations are organized within friendship systems, such that one friend’s exercise behavior, perceived support, and sense of meaning may be linked to the other friend’s psychosocial experiences.

Specifically, this study emphasizes four interrelated relational mechanisms. First, close friends may show peer similarity because they share campus environments, leisure routines, exercise opportunities, and developmental challenges. Second, friends may exert interpersonal influence through companionship, modeling, encouragement, and emotional contagion. Third, shared or socially embedded exercise contexts may provide repeated opportunities for support exchange and relational affirmation. Fourth, meaning in life may be partly co-constructed in friendship dyads, as friends help one another interpret daily experiences, affirm personal value, and develop a sense of direction. These mechanisms are difficult to capture in traditional individual-level analyses because such analyses treat each participant as an independent observation and therefore cannot distinguish whether psychosocial resources are confined within the individual or are also linked across dyad members. From this perspective, the relationship between physical exercise and meaning in life may be reflected not only in an intra-individual pattern, whereby individuals who exercise more report higher levels of social support and meaning in life, but also in an inter-individual pattern, whereby one individual’s exercise or support experiences are associated with their friend’s support experiences and meaning in life.

The actor–partner interdependence model (APIM) provides an appropriate methodological framework for examining this issue (Kenny et al., 2006). Dyadic data analysis theory indicates that responses from members of dyadic relationships—such as intimate partners, friends, parents and children, and spouses—are often interdependent. Therefore, the actor association of individuals’ own predictors with their own outcomes and the partner association of individuals’ predictors with their partners’ outcomes should be estimated simultaneously within the same model. In the present study, actor associations represent the extent to which a student’s own physical exercise and perceived social support are associated with their own meaning in life. Partner associations represent the extent to which one student’s physical exercise and perceived social support are associated with their friend’s perceived support and meaning in life. Thus, the APIM framework allows us to test whether exercise-related psychological benefits are purely individual or whether they are also embedded in cross-partner relational processes within friendship dyads.

Cook and Kenny’s (2005) dyadic data analysis framework provides a classic theoretical and methodological foundation for this approach. Rather than treating members of a dyad as independent observations, this framework emphasizes that dyadic members are linked through shared contexts, reciprocal influence, and relationship-specific processes. Accordingly, dyadic analysis should explicitly account for non-independence and examine how each member’s characteristics are associated not only with their own outcomes, but also with their partner’s outcomes. This logic is directly relevant to close friendship dyads because friends may share daily environments, influence one another’s behaviors, exchange support, and jointly interpret life experiences.

Ledermann et al. (2011) further extended the actor–partner interdependence model to mediation models, forming the Actor–Partner Interdependence Mediation Model (APIMeM). This extension allows researchers to examine whether actor and partner associations operate statistically through mediating variables. In the present study, the APIMeM makes it possible to test whether physical exercise is associated with meaning in life through perceived social support not only within individuals but also across close friends. Specifically, it allows the estimation of actor indirect associations, in which one’s own physical exercise is associated with one’s own meaning in life through social support, and partner indirect associations, in which one dyad member’s physical exercise or social support is associated with the other member’s psychological outcomes. Thus, the APIMeM provides a theoretically appropriate framework for examining whether social support statistically accounts for the association between physical exercise and meaning in life within close friendship dyads.

Recent developmental psychology research has also applied this model to examine the actor and partner associations of social support in parent–child dyads, indicating that this approach is suitable for revealing the interrelations of psychosocial resources, such as social support, within relational systems. Applying this framework to close peer dyads allows the present study to test whether physical exercise, perceived social support, and meaning in life are organized only as individual-level associations or also as interdependent relational processes.

### 1.3. The Present Study

Accordingly, the central research problem of the present study is not whether physical exercise is generally beneficial for college students’ psychological functioning, but whether the association between physical exercise and meaning in life is embedded in close peer relationships. Specifically, this study examines whether physical exercise, perceived social support, and meaning in life show both intra-individual associations and cross-partner associations within close friendship dyads. Compared with traditional individual-level analyses, which can only examine whether a student’s own physical exercise is associated with their own social support and meaning in life, a dyadic approach allows us to examine whether one friend’s exercise behavior and perceived social support are also associated with the other friend’s psychological experiences. In this sense, the present study moves beyond the question of whether exercise is beneficial for individuals and further asks whether exercise-related psychosocial resources may be shared, transmitted, or mutually reinforced within close friendships. These relational processes may include peer similarity in exercise-related routines, interpersonal encouragement and modeling, reciprocal support exchange, emotional contagion, and the co-construction of meaning in life through shared experiences and mutual affirmation.

Based on the theoretical and empirical foundations outlined above, the present study examines the interdependence among physical exercise, social support, and meaning in life among college students from the perspective of close peer dyadic relationships. Accordingly, the key theoretical contribution of this study is to shift the explanation of exercise-related psychological benefits from an isolated individual framework to a relationally embedded dyadic framework. Within this framework, physical exercise is conceptualized as a social context, perceived social support as a relational mechanism, and meaning in life as a psychological outcome that may be linked across close friends. Specifically, this study examines whether individuals’ own physical exercise is associated with their own social support and meaning in life, whether individuals’ physical exercise is also associated with their peers’ social support and meaning in life, and whether social support shows actor-level and partner-level indirect associations between physical exercise and meaning in life. Addressing these questions may deepen understanding of the mechanisms through which physical exercise is associated with mental health development from a relationally embedded perspective.

Specifically, the present study focuses on close peer dyads among college students and uses the actor–partner interdependence model for analysis. Because the two members of each dyad are close friends and do not have clear structural role differences, such as those observed in marital, parent–child, or teacher–student relationships, the present study treats the dyads as indistinguishable. This approach avoids interpreting arbitrary numbering as substantive role differences. The study first examines the non-independence of physical exercise, social support, and meaning in life within dyads. It then investigates actor and partner associations between physical exercise and social support, between social support and meaning in life, and between physical exercise and meaning in life. Finally, it tests whether social support shows actor-level and partner-level statistical indirect associations between physical exercise and meaning in life. Given the cross-sectional design, all paths are interpreted as associations among variables rather than causal effects. The present study proposes the following hypotheses:

**H1.**  *An individual’s physical exercise will be positively associated with their own perceived social support and meaning in life.*

**H2.**  *An individual’s physical exercise will be positively associated with their friend’s perceived social support and meaning in life.*

**H3.**  *Perceived social support will be positively associated with meaning in life at both the actor and partner levels.*

**H4.**  *Perceived social support will statistically account for the indirect association between physical exercise and meaning in life within friendship dyads.*

## 2. Materials and Methods

### 2.1. Study Population

A total of 485 dyads were initially approached to participate in this study, which employed a cross-sectional dyadic survey design at a large public university in Eastern China. Participants were recruited using a friendship-based dyadic recruitment strategy. Students were instructed to participate with a close peer with whom they had regular interaction in daily university life. To ensure that the dyads represented platonic peer friendships rather than romantic relationships, participants were explicitly instructed not to participate with a current romantic partner, such as a boyfriend or girlfriend. Dyad formation was based on naturally occurring peer affiliations rather than random assignment or researcher matching. Both members of each dyad independently completed a questionnaire package, which included a demographic information form and standardized measures of physical exercise, perceived social support, and meaning in life.

For the purposes of this study, a close friendship dyad was defined as a pair of university students who mutually identified each other as close peers and reported regular interaction in daily university life. Dyads were included only when both members confirmed the close friendship. The present study focused exclusively on same-sex friendship dyads; mixed-sex dyads and romantic couples were not included in the final analytical sample. Dyads were excluded if either member reported insufficient familiarity, minimal interaction, or incomplete or unmatched pair information. This operationalization ensured that the dyads reflected naturally occurring close friendships rather than arbitrary pairings for the survey.

Dyads were also excluded if either member had a missing response rate greater than 30%, if critical demographic information was missing, or if response patterns suggested insufficient engagement, such as straight-lining. This exclusion process was implemented as listwise deletion at the dyad level. After these exclusions, the final analytical sample consisted of 415 dyads comprising 830 participants, yielding an effective response rate of 85.58%. All analyses were conducted using these complete dyads.

Among participants, 195 females (47.0%) and 220 males (53.0%) were coded as Member 1; similarly, 195 females (47.0%) and 220 males (53.0%) were coded as Member 2. Ages for Member 1 ranged from 17 to 22 years (M = 19.9, SD = 1.5), and ages for Member 2 ranged from 17 to 23 years (M = 20.0, SD = 1.5). Dyad composition included 200 male–male dyads (48.2%) and 215 female–female dyads (51.8%). No mixed-sex friendship dyads or romantic dyads were included. All dyads reported being close friends, with a mean relationship duration of 1.8 years (SD = 0.9, range = 0.5–4.5 years).

Member 1 and Member 2 labels were arbitrary and were used only for data organization and statistical modeling. They did not represent structurally meaningful roles within the dyad. Therefore, the dyads were treated as indistinguishable friendship dyads in the subsequent dyadic analyses.

### 2.2. Research Tools

Physical exercise levels were assessed using the Physical Exercise Rating Scale-3 (PARS-3; Liang, 1994). This instrument quantifies exercise volume based on three parameters: intensity, duration, and frequency. A global score is calculated using the formula: Score = Intensity × (Duration − 1) × Frequency, with total scores ranging from 0 to 100. Higher scores indicate greater levels of physical exercise. In the current study, the scale demonstrated high internal consistency (Cronbach’s α = 0.895). The physical exercise measure primarily assessed exercise level in terms of intensity, frequency, and duration. Therefore, it should be understood as an indicator of overall exercise involvement rather than a comprehensive measure of the qualitative, social, or experiential characteristics of physical exercise.

Social support was measured using the Adolescent Social Support Scale (Ye & Dai, 2008), which has been validated in the Chinese cultural context. The scale comprises 17 items assessing three dimensions: emotional support, instrumental support, and informational support. Items were rated on a 5-point Likert scale (1 = strongly disagree to 5 = strongly agree), with higher aggregate scores indicating higher levels of perceived social support. This scale assesses general perceived social support rather than exercise-specific social support. Therefore, in the present study, social support refers to students’ overall perception of available emotional, instrumental, and informational support in their social relationships, rather than support specifically related to physical exercise. The scale showed excellent internal consistency in the current sample (Cronbach’s α = 0.951).

Meaning in life was assessed using the Chinese version of the Meaning in Life Questionnaire (MLQ; Steger, 2009), as revised by Liu and Gan (2010). This 10-item scale evaluates the extent to which individuals feel their lives have meaning (Presence of Meaning) and their motivation to find meaning (Search for Meaning). Items were rated on a 7-point Likert scale (1 = absolutely untrue to 7 = absolutely true). For the purposes of this study, the total score was calculated to reflect the overall sense of meaning in life. The scale demonstrated high reliability in the current sample (Cronbach’s α = 0.924).

### 2.3. Data Processing and Statistical Analysis

All data processing and statistical analyses were conducted in R version 4.5.2. Data screening was first performed to identify missing responses, incomplete dyad matching, invalid pair information, and potentially careless response patterns. Dyads were excluded if either member had a missing response rate greater than 30%, if critical demographic information was missing, if response patterns suggested insufficient engagement, or if pair information was incomplete or unmatched.

Descriptive statistics, including means, standard deviations, ranges, and skewness, were calculated for physical exercise, social support, and meaning in life. Pearson correlation analyses were then conducted to examine bivariate associations among the study variables for both dyad members. Intraclass correlation coefficients (ICCs) were calculated to assess within-dyad non-independence and to determine whether dyadic modeling was appropriate.

Because the study examined whether one member’s physical exercise was associated with both their own and their friend’s psychosocial outcomes, dyadic analyses were specified as the primary analytic strategy. Given that the two members of each friendship dyad were self-identified close peers without structurally distinct roles, the dyads were treated as indistinguishable.

An Actor–Partner Interdependence Mediation Model (APIMeM) was estimated, with physical exercise specified as the predictor, social support as the statistical linking variable, and meaning in life as the outcome. The model estimated actor and partner associations from physical exercise to social support, actor and partner associations from social support to meaning in life, direct actor and partner associations from physical exercise to meaning in life, and indirect associations through social support.

A constrained indistinguishable APIMeM was specified as the primary model, in which corresponding actor and partner paths for the two dyad members were constrained to equality. To evaluate whether these equality constraints were acceptable, this model was compared with an unconstrained APIMeM in which corresponding paths were freely estimated. Model comparison was based on the chi-square difference test, AIC, and BIC. Model fit was evaluated using χ^2^, df, CFI, TLI, RMSEA, and SRMR. Indirect associations were tested using bootstrap confidence intervals. Because the study was cross-sectional, indirect associations were interpreted as statistical indirect associations rather than causal indirect associations.

## 3. Results

### 3.1. Preliminary Analyses

The final sample consisted of 415 peer dyads, including 830 college students. Because the two members of each dyad did not occupy structurally distinct roles, the dyads were treated as indistinguishable in the subsequent APIMeM analyses. Member 1 and Member 2 were arbitrary labels used only for data organization and did not represent theoretically meaningful roles within the dyad. Descriptive statistics and intraclass correlations are presented in Table 1. On average, Member 1 reported a physical exercise score of 22.38, a social support score of 49.43, and a meaning in life score of 35.34. Member 2 reported a physical exercise score of 19.41, a social support score of 49.12, and a meaning in life score of 34.33.

Intraclass correlations were calculated to evaluate within-dyad non-independence. The ICCs were 0.101 for physical exercise, 0.188 for social support, and 0.253 for meaning in life, indicating that 10.1%, 18.8%, and 25.3% of the variance in these variables, respectively, was attributable to between-dyad differences. These results suggest non-negligible within-dyad similarity and support the use of dyadic analyses.

Bivariate correlations among the main study variables are presented in Table 2. Physical exercise, social support, and meaning in life were generally positively correlated both within and across dyad members. At the intra-individual level, physical exercise was positively correlated with social support and meaning in life for both Member 1 and Member 2. Cross-member correlations were also significant, indicating meaningful interdependence between peer partners. Residual correlations between dyad members were estimated in the APIMeM to account for unmeasured shared variance. The estimated residual correlation for social support was r = 0.18, *p* < 0.001, and that for meaning in life was r = 0.25, *p* < 0.001. These results indicate that, after accounting for actor and partner associations, meaningful shared dyadic variance remained in social support and meaning in life.

To assess the potential for common method bias, Harman’s single-factor test was conducted by entering all items of physical exercise, social support, and meaning in life into an exploratory factor analysis. The results indicated that a single factor accounted for 28.97% of the total variance, which is below the commonly used threshold of 40%, suggesting that common method variance was unlikely to substantially bias the findings.

### 3.2. Actor–Partner Association Between Physical Exercise and Social Support

**Figure 1:**
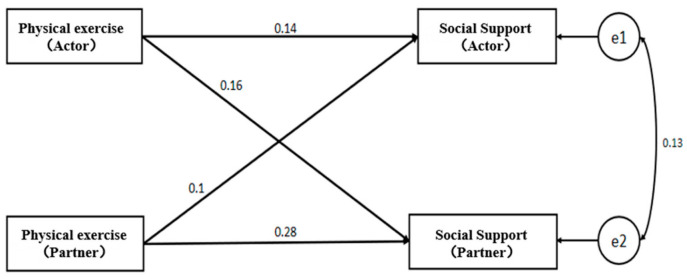
Actor–Partner Interdependence Model of Physical Exercise and Social Support.

An APIM was specified to examine the association between physical exercise and social support within friendship dyads. Each member’s physical exercise was specified to predict both their own social support (actor association) and their partner’s social support (partner association). The two members’ physical exercise variables were allowed to covary, and the residuals of the two social support variables were also allowed to covary to account for dyadic interdependence. The model structure and standardized path estimates are presented in Figure 1. The APIM showed the following fit indices: χ^2^/df = 4.854, RMSEA = 0.091, NFI = 0.933, IFI = 0.946, and CFI = 0.942. Path analysis showed significant actor associations: actor physical exercise was positively associated with actor social support (β = 0.136, *p* = 0.005), and partner physical exercise was positively associated with partner social support (β = 0.275, *p* < 0.001). In addition, significant partner associations were observed: actor physical exercise was positively associated with partner social support (β = 0.161, *p* < 0.001), and partner physical exercise was positively associated with actor social support (β = 0.099, *p* = 0.034). As shown in Figure 1, both actor and partner paths were significant, indicating that physical exercise was associated with social support not only within individuals, but also across friendship partners.

### 3.3. Actor–Partner Association Between Social Support and Meaning in Life

**Figure 2:**
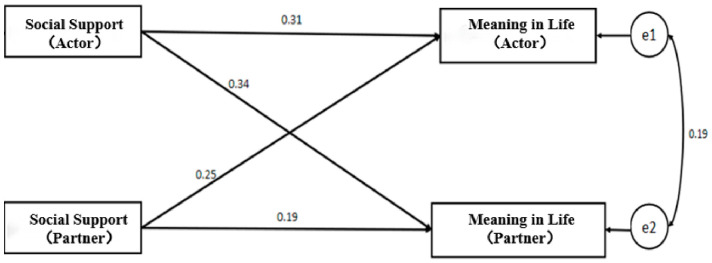
Actor–Partner Interdependence Model of Social Support and Meaning in Life.

A second APIM was specified to examine the association between social support and meaning in life. Each member’s social support was specified to predict both their own meaning in life (actor association) and their partner’s meaning in life (partner association). The two members’ social support variables were allowed to covary, and the residuals of the two meaning-in-life variables were also allowed to covary. The model structure and standardized path estimates are shown in Figure 2. The APIM showed the following fit indices: χ^2^/df = 3.098, RMSEA = 0.046, NFI = 0.984, IFI = 0.989, and CFI = 0.988. Path analysis showed significant actor associations: actor social support was positively associated with actor meaning in life (β = 0.313, *p* < 0.001), and partner social support was positively associated with partner meaning in life (β = 0.194, *p* < 0.001). Significant partner associations were also observed: actor social support was positively associated with partner meaning in life (β = 0.343, *p* < 0.001), and partner social support was positively associated with actor meaning in life (β = 0.252, *p* < 0.001). As illustrated in Figure 2, social support showed significant actor and partner associations with meaning in life, suggesting that perceived support was linked to meaning in life both intrapersonally and interpersonally.

### 3.4. Actor–Partner Association Between Physical Exercise and Meaning in Life

**Figure 3:**
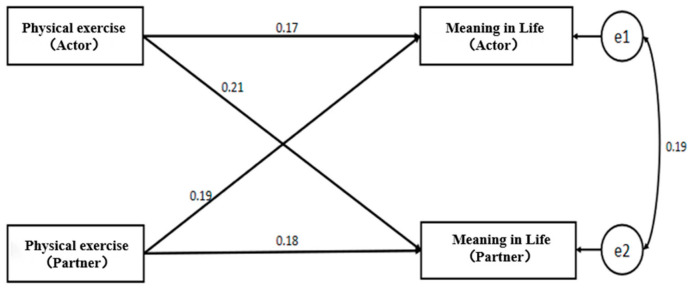
Actor–Partner Interdependence Model of Physical Exercise and Meaning in Life.

An APIM was then estimated to examine the association between physical exercise and meaning in life at both the actor and partner levels. In this model, each member’s physical exercise was specified to predict both members’ meaning in life. Corresponding exogenous variables and parallel endogenous residuals were allowed to covary where appropriate. The model structure and standardized path estimates are displayed in Figure 3. The APIM showed the following fit indices: χ^2^/df = 3.098, RMSEA = 0.096, NFI = 0.944, IFI = 0.955, and CFI = 0.952. Path analysis showed significant actor associations: actor physical exercise was positively associated with actor meaning in life (β = 0.169, *p* < 0.001). In addition, partner physical exercise was positively associated with partner meaning in life (β = 0.179, *p* < 0.001). Significant partner associations were also observed: actor physical exercise was positively associated with partner meaning in life (β = 0.209, *p* < 0.001), and partner physical exercise was positively associated with actor meaning in life (β = 0.188, *p* < 0.001). As shown in Figure 3, physical exercise was associated with meaning in life at both the actor and partner levels, indicating that exercise-related psychological benefits may extend across close friendship dyads.

### 3.5. Model Comparison Between the Unconstrained and Constrained APIMeM

Because the dyads consisted of peers without structurally distinct roles, a constrained indistinguishable APIMeM was specified as the primary model. In the present study, Member 1 and Member 2 were labels used for data organization rather than theoretically meaningful roles. Unlike parent–child, teacher–student, or romantic partner dyads, the two members of each peer dyad did not differ in any predefined relational status. To evaluate the tenability of the equality constraints, this model was compared with an unconstrained APIMeM. This comparison served as the omnibus test of indistinguishability, assessing whether constraining the corresponding parameters of the two dyad members to equality significantly worsened model fit.

As shown in Table 3, the unconstrained model was saturated, χ^2^(0) = 0.00, CFI = 1.000, TLI = 1.000, RMSEA = 0.000, and SRMR = 0.000. Therefore, this model served as a reference model rather than a substantive test of global model fit. The constrained indistinguishable APIMeM showed acceptable fit to the data, χ^2^(6) = 12.81, *p* = 0.046, CFI = 0.973, TLI = 0.933, RMSEA = 0.052, 90% CI [0.007, 0.092], and SRMR = 0.028.

The omnibus chi-square difference test of indistinguishability indicated a small but statistically significant decrease in fit after imposing equality constraints, Δχ^2^(6) = 12.81, *p* = 0.046. Thus, the statistical test did not provide full support for complete empirical indistinguishability. However, this result was interpreted in light of the theoretical nature of the dyads and the overall model comparison indices. Although Member 1 and Member 2 showed some descriptive differences, including differences in physical exercise scores, these differences should not be interpreted as evidence of substantively distinguishable dyadic roles because the assignment of members was arbitrary and not based on any theoretically meaningful characteristic. Moreover, the AIC values were highly similar between the two models, whereas the BIC favored the more parsimonious constrained model. Therefore, based on theoretical indistinguishability, acceptable absolute fit, model parsimony, and lower BIC, the constrained indistinguishable APIMeM was retained as the primary model. The unconstrained model was examined as a sensitivity analysis.

### 3.6. Actor and Partner Association in the Constrained APIMeM

The constrained indistinguishable APIMeM showed significant actor and partner associations among physical exercise, social support, and meaning in life. The path estimates are presented in Table 4. Physical exercise was positively associated with social support at both the actor level (B = 0.150, SE = 0.026, 95% CI [0.097, 0.200], *p* < 0.001) and the partner level (B = 0.095, SE = 0.026, 95% CI [0.043, 0.144], *p* < 0.001). These findings indicate that individuals with higher levels of physical exercise tended to report higher levels of their own social support, and their peer partners also tended to report higher levels of social support. Social support was positively associated with meaning in life at both the actor level (B = 0.174, SE = 0.026, 95% CI [0.122, 0.223], *p* < 0.001) and the partner level (B = 0.206, SE = 0.025, 95% CI [0.159, 0.257], *p* < 0.001). Thus, both one’s own social support and one’s peer partner’s social support were positively associated with meaning in life. The direct effects of physical exercise on meaning in life remained significant at both the actor level (B = 0.055, SE = 0.017, 95% CI [0.021, 0.087], *p* = 0.001) and the partner level (B = 0.067, SE = 0.018, 95% CI [0.033, 0.105], *p* < 0.001). These results suggest that physical exercise was directly associated with meaning in life even after accounting for social support.

The model explained 6.5% of the variance in SS(actor) (R^2^ = 0.065) and 6.1% of the variance in SS(partner) (R^2^ = 0.061), indicating small explanatory power for social support. The model explained 20.4% of the variance in ML(actor) (R^2^ = 0.204) and 20.2% of the variance in ML(partner) (R^2^ = 0.202), indicating moderate explanatory power for meaning in life. Taken together, the model accounted for a modest proportion of variance in social support and a more substantial, moderate proportion of variance in meaning in life.

### 3.7. Indirect Association of Physical Exercise with Meaning in Life Through Social Support

Bootstrap analyses were conducted to test the indirect association of physical exercise with meaning in life through social support. The statistical indirect association estimates are presented in Table 5.

As shown in Table 5, all specific indirect associations reached statistical significance. For actor pathways, one’s own physical exercise was indirectly associated with one’s own meaning in life through one’s own social support (B = 0.026, SE = 0.006, 95% CI [0.015, 0.039], *p* < 0.001). A second actor-related pathway also emerged through the peer partner’s social support (B = 0.020, SE = 0.006, 95% CI [0.009, 0.033], *p* = 0.001).

For partner pathways, the peer partner’s physical exercise was indirectly associated with one’s own meaning in life via one’s own social support (B = 0.017, SE = 0.005, 95% CI [0.008, 0.028], *p* = 0.001). In addition, an indirect pathway was observed through the partner’s own social support (B = 0.031, SE = 0.007, 95% CI [0.019, 0.045], *p* < 0.001).

When these specific pathways were combined, the total actor indirect association was significant (B = 0.046, SE = 0.008, 95% CI [0.030, 0.063], *p* < 0.001). The total partner indirect association showed a comparable pattern (B = 0.047, SE = 0.008, 95% CI [0.032, 0.065], *p* < 0.001). These findings indicate that perceived social support statistically accounted for both actor and partner associations between physical exercise and meaning in life.

**Figure 4:**
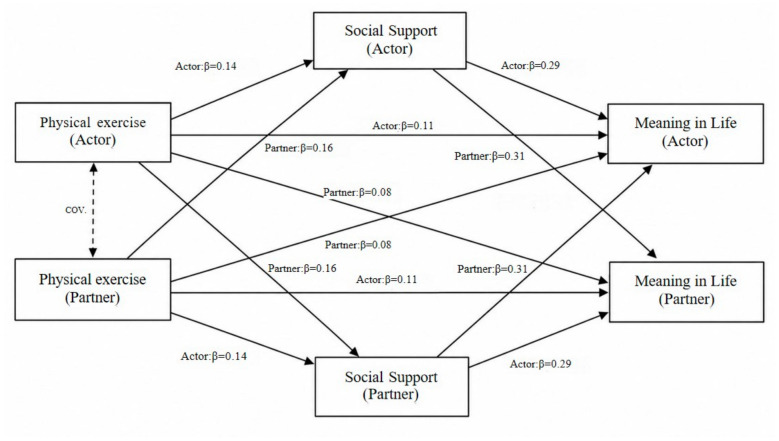
Actor–Partner Interdependence Mediation Model.

The overall associations remained significant: the total actor association was B = 0.100, SE = 0.018, 95% CI [0.066, 0.135], *p* < 0.001, and the total partner association was B = 0.115, SE = 0.020, 95% CI [0.078, 0.154], *p* < 0.001. Because the direct associations between physical exercise and meaning in life remained significant after social support was included, the results support partial statistical indirect associations rather than full mediation, as illustrated in Figure 4.

### 3.8. Sensitivity Analysis of the Indirect Association Between Physical Exercise and Meaning in Life Through Social Support

As a sensitivity analysis, the unconstrained APIMeM was examined. Because the unconstrained model was saturated, it did not provide interpretable global fit indices. The path estimates from the unconstrained model are presented in Table 6.

Overall, the pattern of findings was broadly consistent with that of the constrained model. Physical exercise was positively associated with social support both within individuals and across dyad members. Specifically, PE1 was positively associated with SS1 (B = 0.094, *p* = 0.005), and PE2 was positively associated with SS2 (B = 0.218, *p* < 0.001). Cross-member associations were also significant: PE2 was positively associated with SS1 (B = 0.125, *p* = 0.001), and PE1 was positively associated with SS2 (B = 0.070, *p* = 0.035).

Similarly, social support was positively associated with meaning in life both within and across dyad members. SS1 was positively associated with ML1 (B = 0.226, *p* < 0.001), and SS2 was positively associated with ML2 (B = 0.122, *p* = 0.001). Cross-member associations were also significant: SS2 was positively associated with ML1 (B = 0.170, *p* < 0.001), and SS1 was positively associated with ML2 (B = 0.248, *p* < 0.001).

The direct paths from physical exercise to meaning in life were less consistent in the unconstrained model. PE1 was significantly associated with ML1 (B = 0.059, *p* = 0.015), and PE1 was also significantly associated with ML2 (B = 0.083, *p* = 0.001). However, PE2 was not significantly associated with ML1 (B = 0.049, *p* = 0.080) or ML2 (B = 0.053, *p* = 0.065). This pattern suggests that the indirect pathways through perceived social support were more robust than some of the direct associations between physical exercise and meaning in life.

Therefore, the results of the unconstrained model were treated as a sensitivity analysis, whereas the constrained indistinguishable APIMeM was retained as the primary model because it was theoretically appropriate for peer dyads, showed acceptable fit, and had a lower BIC.

## 4. Discussion

The present study examined the associations among physical exercise, social support, and meaning in life among college students from the perspective of close peer dyadic relationships. Overall, the results supported the study hypotheses. Physical exercise was positively associated not only with individuals’ own social support and meaning in life, but also with their peers’ social support and meaning in life. These findings indicate that the relationship between physical exercise and meaning in life among college students is not merely an intra-individual psychological process; rather, it may also be embedded in the supportive social context provided by close peer relationships. Importantly, given the cross-sectional design, the partner effects should be interpreted as cross-partner associations within friendship dyads rather than as evidence of direct interpersonal influence. In addition, social support showed significant actor-level and partner-level statistical indirect associations between physical exercise and meaning in life.

These results should also be interpreted in relation to how physical exercise was measured in this study, namely, as an overall exercise level based on intensity, frequency, and duration. Therefore, higher measured levels of physical exercise were associated with greater perceived social support and meaning in life within friendship dyads. This does not imply that only high-intensity, high-frequency, or long-duration exercise is meaningful. Light or moderate activities, such as walking with friends, may also provide relational and psychological benefits. However, the present study was not designed to examine differences between exercise intensity levels or to capture the qualitative, social, or experiential aspects of physical exercise. In other words, the psychological meaning of physical exercise observed here may partly derive from its relational attributes, such as opportunities for interaction, companionship, encouragement, and shared experiences, while acknowledging that such relational and meaningful experiences may occur across a range of exercise intensities and frequencies.

The study found that physical exercise was positively associated with individuals’ own perceived social support and meaning in life. This finding is consistent with existing research on physical exercise and positive psychological functioning, suggesting that physical exercise may be associated with higher levels of social connectedness, psychological resources, and positive adaptation (Pan et al., 2025; K. Wang et al., 2022). Compared with previous individual-level studies, the present study further suggests that these associations may also be embedded in close friendship dyads. That is, physical exercise, perceived social support, and meaning in life were not only associated within individuals, but also modestly linked across close friends. In college life, physical exercise often occurs in social contexts, including physical education classes, dormitory life, club activities, exercise with friends, and campus leisure activities (X. Zhou et al., 2025). Therefore, higher levels of physical exercise may reflect more opportunities to participate in shared activities, receive peer encouragement, and experience social connectedness (Y. Wang & Chen, 2025). At the same time, physical exercise may also be associated with a sense of competence, self-efficacy, physical vitality, and daily structure, all of which may help students develop more positive life evaluations and clearer experiences of goals (Goldbeck & Klink, 2022).

In addition, significant partner associations, as well as significant actor associations, were observed between social support and meaning in life. Social support can provide individuals with a sense of acceptance, belonging, and value affirmation, thereby helping them integrate daily experiences into life narratives characterized by direction and purpose (Lambert et al., 2013; Stillman et al., 2009). The findings of the present study further indicate that this association is not confined to the intra-individual level. Specifically, higher perceived social support in one peer was associated with higher meaning in life in the other peer. This partner association should be understood as evidence of modest dyadic interdependence within a shared friendship context, rather than as evidence that one friend’s perceived support directly caused changes in the other friend’s meaning in life (Lambert et al., 2013). Companionship, emotional sharing, encouragement, and shared experiences between close friends may constitute relational resources that enable both parties to more readily experience coherence, direction, and value in life (Lambert et al., 2013; Stillman et al., 2009). From a dyadic perspective, meaning in life may also be partly co-constructed in friendship pairs. Close friends often discuss daily experiences, provide emotional validation, and help each other interpret challenges and goals. Through these repeated interactions, one member’s perceived support may contribute to a relational climate in which both members feel valued, understood, and more capable of locating meaning in everyday life.

The partner association of physical exercise indicates that physical exercise may have psychosocial significance beyond the individual level. The results showed that individuals’ physical exercise was associated not only with their own social support and meaning in life, but also with their peers’ social support and meaning in life (Jiang et al., 2025). However, because the present study used a cross-sectional design, this finding should not be interpreted as evidence that one student’s physical exercise directly influenced their friend’s social support or meaning in life. Rather, the partner association suggests that physical exercise may be linked across dyad members through a shared relational context within close friendships. One plausible explanation for this finding is that physical exercise may reflect or promote a more interactive peer relational context (Shi et al., 2026). Students who participate more actively in exercise may be more likely to invite friends to engage in activities together, share exercise experiences, provide emotional encouragement, and develop more regular patterns of shared activity in daily life (Shi et al., 2026; X. Zhou et al., 2025). Several psychological mechanisms may explain why one member’s physical exercise is associated with the other member’s perceived social support or meaning in life. First, behavioral modeling may be involved. When one friend regularly participates in physical exercise, this behavior may provide a visible model of active coping, self-regulation, and health-oriented living. The other friend may observe this behavior and gradually perceive exercise as a meaningful, attainable, and socially valued activity. Second, shared physical activities may strengthen the partner’s perceived support. Friends who exercise together, discuss exercise plans, or participate in the same campus sport contexts may have more opportunities for companionship, cooperation, and mutual assistance. These repeated interactions can increase the partner’s sense of being accompanied and supported. Third, verbal encouragement may be another important mechanism. A physically active student may invite a friend to exercise, encourage persistence, provide feedback, or express concern for the friend’s health and well-being. Such supportive communication may directly enhance the friend’s perceived social support. Fourth, emotional reinforcement and emotional contagion may also contribute to partner associations. Exercise-related positive emotions, such as vitality, confidence, and enjoyment, may shape the emotional atmosphere of the friendship and indirectly influence the partner’s psychological experience. It is also important to consider the nature of the social support measure used in the present study. The Adolescent Social Support Scale assesses general perceived social support, including emotional, instrumental, and informational support, rather than exercise-specific social support. Therefore, the observed associations involving social support should be interpreted as reflecting students’ overall perception of support within their social relationships, not support specifically tied to exercise participation. In this sense, the partner associations may partly reflect the general quality of close friendship relationships or the broader supportive climate within the dyad. For example, a student who exercises more may also be embedded in a more active, connected, and supportive friendship context, and this overall relational quality may be associated with the friend’s perceived support and meaning in life. Thus, although shared physical exercise, exercise-related encouragement, and behavioral modeling remain plausible mechanisms, the present data cannot determine whether the observed partner associations are driven specifically by exercise-related support or by general perceived social support. Future studies should include exercise-specific social support measures to distinguish general relationship quality from support processes directly related to physical exercise.

These mechanisms suggest that physical exercise in friendship dyads should not be understood merely as an individual health behavior. Rather, it may operate as a relationally embedded activity through which friends model behaviors, share experiences, encourage one another, and create a supportive interpersonal climate. This interpretation helps explain how one member’s physical exercise may be linked to the other member’s perceived social support or meaning in life. The observed partner associations may therefore reflect not only statistical non-independence, but also meaningful interpersonal processes within close friendships. Although these interactions do not necessarily imply unidirectional influence, they may be associated with stronger peer connectedness and a more supportive relational climate. Therefore, the partner associations observed in the present study should not be interpreted simply as evidence that one person’s exercise directly increases a friend’s meaning in life. Rather, they should be understood as reflecting coordinated associations among physical exercise, social support, and meaning-related experiences within close peer relationships. Given the cross-sectional design, these mechanisms should be regarded as theoretically plausible explanations rather than causal conclusions. Future longitudinal, observational, or experience-sampling studies could further examine whether behavioral modeling, shared exercise participation, verbal encouragement, and emotional contagion mediate the partner-level associations among physical exercise, perceived social support, and meaning in life.

The mediation model results indicated that social support served as an important statistical bridge linking physical exercise and meaning in life (Ledermann et al., 2011). All specific indirect associations reached statistical significance, and both the total actor indirect association and the total partner indirect association were significant (Ledermann et al., 2011). This finding suggests that the association between physical exercise and meaning in life may be partly reflected through social support as a relational resource. Notably, after social support was included in the model, the direct effect of physical exercise on meaning in life remained significant, indicating that social support did not fully explain this association. Physical exercise may also be associated with meaning in life through other pathways, such as self-efficacy, emotion regulation, physical vitality, life satisfaction, self-control, or a sense of purpose (G.-Y. Zhou et al., 2023). However, the sensitivity analysis further suggested that the direct effects should be interpreted with caution. Although the constrained indistinguishable Actor–Partner Interdependence Mediation Model (APIMeM) showed significant actor and partner direct effects of physical exercise on meaning in life, some direct paths from physical exercise to meaning in life were not significant in the unconstrained model. In contrast, the paths from physical exercise to social support and from social support to meaning in life were more stable across models (Jiang et al., 2025). This pattern indicates that, compared with the direct association between physical exercise and meaning in life, the indirect pathway through social support may have greater explanatory power and robustness. In theoretical terms, this finding further supports the view that the psychological significance of exercise among college students may be partly transmitted through relational resources. Exercise may create or reflect supportive friendship contexts, and these contexts may be closely linked to how students experience value, belonging, and meaning in life.

From a practical perspective, these findings extend previous individual-level studies by suggesting that college physical exercise and mental health programs may benefit from considering peer relational contexts. Previous studies have mainly emphasized that physical exercise is associated with individuals’ own psychological resources and positive adaptation. The present dyadic findings further suggest that exercise-related experiences may also be modestly connected across close friends. Therefore, universities may consider encouraging friend-based exercise participation, peer-supported physical activity, and small-group campus exercise. Such approaches may help create supportive relational contexts in which students experience companionship, encouragement, belonging, and shared purpose. However, given the modest partner associations and the cross-sectional design, these implications should be interpreted cautiously and should not be understood as evidence of causal interpersonal influence.

## 5. Limitations

The present study has several limitations. First, the cross-sectional design cannot determine the temporal order or causal direction among the variables. Physical exercise may be associated with higher levels of social support and meaning in life, but students with higher social support or a stronger sense of meaning in life may also be more willing to participate in physical exercise. Therefore, the observed partner associations should be interpreted as statistical links within friendship dyads rather than as evidence of direct interpersonal influence. Similarly, the ordering of physical exercise, perceived social support, and meaning in life should be understood as theoretically driven rather than empirically established. The present data cannot rule out alternative structural configurations, such as the possibility that meaning in life or perceived social support may also be associated with a greater willingness to participate in physical exercise. Future research should adopt longitudinal follow-up, cross-lagged, diary, experimental, or intervention designs to examine the dynamic relationships among physical exercise, social support, and meaning in life.

Second, the present study measured individuals’ overall perceived social support rather than support specifically received from the paired peer. Therefore, partner social support should be understood as the peer’s self-reported overall level of social support rather than the support actually provided by that peer to the other member within the dyadic relationship. Future research should further distinguish among overall social support, friend-specific support, and actual support exchange.

Third, all variables were measured using self-report measures, which may involve common method bias. Future research could incorporate objective exercise data, peer reports, behavioral observations, and ecological momentary assessment.

Fourth, the sample was drawn from one university in Eastern China and included only close peer dyads. Therefore, whether the findings can be generalized to different regions, different types of universities, cross-sex friendships, roommate relationships, sports teams, or other cultural contexts requires further examination.

Fifth, the present study used the total score of meaning in life, although presence of meaning and search for meaning may have different psychological implications. Future research could examine their actor and partner associations in peer relationships separately.

## 6. Conclusions

This study examined the dyadic associations among physical exercise, perceived social support, and meaning in life in friendship dyads of university students. Using the Actor–Partner Interdependence Mediation Model, the findings showed that physical exercise was positively associated with meaning in life at both the actor and partner levels, and that perceived social support showed significant actor-level and partner-level statistical indirect associations. These results suggest that the association between physical exercise and meaning in life is not only an individual-level psychological process but may also be embedded in close peer relational contexts.

The main contribution of this study is that it extends previous individual-level research by showing modest cross-partner associations among physical exercise, perceived social support, and meaning in life within friendship dyads. This finding highlights the usefulness of the APIM approach for examining relational interdependence among college students. Practically, the results suggest that university physical exercise and mental health programs may benefit from incorporating peer-based elements, such as friend-based exercise participation, peer encouragement, and small-group campus sports. Moreover, these findings imply that interventions targeting student well-being could be enhanced by leveraging peer networks, promoting collaborative exercise programs, and fostering social support structures within campus communities.

Several limitations should be noted. Because the study used a cross-sectional design and self-report measures, the findings should not be interpreted as evidence of causal interpersonal influence. In addition, social support was measured as overall perceived support rather than friend-specific support, and the sample was drawn from one university in Eastern China. Future studies should use longitudinal, cross-lagged, diary, or intervention designs; include friend-specific support and behavioral indicators of exercise; and examine more diverse dyadic samples. Extending research across different cultural and institutional contexts could inform policies and programs aimed at integrating physical activity with psychosocial development, mental health promotion, and peer support initiatives in higher education. Overall, this study provides a relational perspective for understanding the psychological significance of physical exercise among university students.

## Figures and Tables

**Table 1:** Descriptive statistics and intraclass correlations for study variables.

Variable	Member	*n*	M	SD	Min	Max	Skewness	ICC
PE	Member 1	415	22.38	19.65	0	100	1.5	0.101
PE	Member 2	415	19.41	17.54	0	100	1.64	0.101
SS	Member 1	415	49.43	13.68	22	76	−0.13	0.188
SS	Member 2	415	49.12	13.9	22	79	−0.01	0.188
ML	Member 1	415	35.34	10.71	14	57	−0.15	0.253
ML	Member 2	415	34.33	10.86	12	56	0.02	0.253

Note. Member 1 and Member 2 were arbitrary labels used only for data organization and did not represent theoretically distinct roles within the peer dyads. ICC = intraclass correlation coefficient; PE = physical exercise; SS = social support; ML = meaning in life.

**Table 2:** Correlations among physical exercise, social support, and meaning in life.

Variable	1	2	3	4	5	6
1. PE1	—					
2. SS1	0.153 **	—				
3. ML1	0.189 ***	0.360 ***	—			
4. PE2	0.108 *	0.175 ***	0.205 ***	—		
5. SS2	0.129 **	0.187 ***	0.311 ***	0.285 ***	—	
6. ML2	0.228 ***	0.379 ***	0.254 ***	0.200 ***	0.258 ***	—

Note. PE = physical exercise; SS = social support; ML = meaning in life. PE1, SS1, and ML1 refer to Member 1; PE2, SS2, and ML2 refer to Member 2. Member labels were arbitrary. The significance of each correlation coefficient is indicated by the number of asterisks: * *p* < 0.05, ** *p* < 0.01, *** *p* < 0.001.

**Table 3:** Model comparison between unconstrained and constrained APIMeM.

Model	χ^2^	df	*p*	CFI	TLI	RMSEA [90% CI]	SRMR	AIC	BIC
Unconstrained	0	0	—	1	1	0.000 [0.000, 0.000]	0	19,997.26	20,106.02
Constrained indistinguishable	12.81	6	0.046	0.973	0.933	0.052 [0.007, 0.092]	0.028	19,998.07	20,082.67

Note. APIMeM = Actor–Partner Interdependence Mediation Model; χ^2^ = chi-square; df = degrees of freedom; *p* = significance value; CFI = Comparative Fit Index; TLI = Tucker–Lewis Index; RMSEA = Root Mean Square Error of Approximation; CI = confidence interval; SRMR = Standardized Root Mean Square Residual; AIC = Akaike Information Criterion; BIC = Bayesian Information Criterion. The unconstrained APIMeM was saturated and was used as a reference model. The constrained indistinguishable APIMeM was retained as the primary model because it was theoretically appropriate for indistinguishable peer dyads, showed acceptable fit, and had a lower BIC.

**Table 4:** Path estimates from the constrained indistinguishable APIMeM.

Path	Effect Type	B	SE	95% CI	*p*	Standardized β
PE → SS	actor association	0.15	0.026	[0.097, 0.200]	<0.001	0.191–0.212
PE → SS	partner association	0.095	0.026	[0.043, 0.144]	<0.001	0.121–0.136
SS → ML	actor association	0.174	0.026	[0.122, 0.223]	<0.001	0.220–0.224
SS → ML	partner association	0.206	0.025	[0.159, 0.257]	<0.001	0.263–0.264
PE → ML	Actor direct association	0.055	0.017	[0.021, 0.087]	0.001	0.088–0.100
PE → ML	Partner direct association	0.067	0.018	[0.033, 0.105]	<0.001	0.110–0.122

Note. PE = physical exercise; SS = social support; ML = meaning in life. Actor association refers to associations between an individual’s own predictor and their own outcome. Partner association refers to associations between one dyad member’s predictor and the other member’s outcome. Equality constraints were imposed on corresponding unstandardized paths because dyads were treated as indistinguishable. Standardized coefficients are presented as ranges because standardized estimates differed slightly across dyad members due to differences in observed variances.

**Table 5:** Bootstrap statistical indirect association from the constrained indistinguishable APIMeM.

Effect	Path	B	SE	95% CI	*p*	Standardized β
Actor via own social support	PE_i_ → SS_i_ → ML_i_	0.026	0.006	[0.015, 0.039]	<0.001	0.048
Actor via partner social support	PE_i_ → SS_j_ → ML_i_	0.02	0.006	[0.009, 0.033]	0.001	0.032
Partner via own social support	PE_j_ → SS_i_ → ML_i_	0.017	0.005	[0.008, 0.028]	0.001	0.027
Partner via partner social support	PE_j_ → SS_j_ → ML_i_	0.031	0.007	[0.019, 0.045]	<0.001	0.056
Total actor indirect association	PE_i_ → SS_i_/SS_j_ → ML_i_	0.046	0.008	[0.030, 0.063]	<0.001	0.079
Total partner indirect association	PE_j_ → SS_i_/SS_j_ → ML_i_	0.047	0.008	[0.032, 0.065]	<0.001	0.083
Total actor association	PE_i_ → ML_i_	0.1	0.018	[0.066, 0.135]	<0.001	0.179
Total partner association	PE_j_ → ML_i_	0.115	0.02	[0.078, 0.154]	<0.001	0.193

Note. PE_i_, SS_i_, and ML_i_ refer to the individual’s own physical exercise, social support, and meaning in life. PE_j_ and SS_j_ refer to the peer partner’s physical exercise and social support. Confidence intervals were estimated using bootstrap procedures. Because the study used cross-sectional data, statistical indirect association should be interpreted as statistical indirect associations rather than evidence of causal mediation.

**Table 6:** Path estimates from the unconstrained APIMeM.

Path	B	SE	95% CI	*p*	Standardized β
PE1 → SS1	0.094	0.034	[0.028, 0.160]	0.005	0.135
PE2 → SS1	0.125	0.038	[0.052, 0.199]	0.001	0.161
PE2 → SS2	0.218	0.037	[0.144, 0.291]	<0.001	0.275
PE1 → SS2	0.07	0.033	[0.005, 0.135]	0.035	0.099
PE1 → ML1	0.059	0.024	[0.011, 0.106]	0.015	0.108
PE2 → ML1	0.049	0.028	[−0.006, 0.104]	0.08	0.08
SS1 → ML1	0.226	0.035	[0.157, 0.295]	<0.001	0.288
SS2 → ML1	0.17	0.036	[0.100, 0.239]	<0.001	0.22
PE2 → ML2	0.053	0.028	[−0.003, 0.108]	0.065	0.085
PE1 → ML2	0.083	0.025	[0.035, 0.132]	0.001	0.151
SS2 → ML2	0.122	0.036	[0.051, 0.193]	0.001	0.156
SS1 → ML2	0.248	0.036	[0.178, 0.318]	<0.001	0.312

Note. PE = physical exercise; SS = social support; ML = meaning in life. The unconstrained model was saturated and was used as a sensitivity analysis. Member 1 and Member 2 were arbitrary labels and did not represent theoretically distinct dyadic roles.

## Data Availability

The data presented in this study are available on request from the corresponding author.
